# Virtual stress testing: Analyzing endocrine, metabolic, cardiovascular, and psychological responses to the TSST-VR

**DOI:** 10.1016/j.ynstr.2025.100760

**Published:** 2025-09-13

**Authors:** Eva Fellinger, Tom Brandt, Andrea Schittenhelm, Eric Quarg, Matthias Pröll, Gregor Domes, Annette Schmidt

**Affiliations:** a dtec.bw, NextGenerationEU Project Smart Health Lab, University of the Bundeswehr Munich, Germany; b University of the Bundeswehr Munich, Germany, Institute of Sport Sciences, Germany; c Biological and Clinical Psychology, Department of Psychology, University of Trier, Johanniterufer 15, 54290, Trier, Germany; d Institute for Cognitive and Affective Neuroscience, University of Trier, Trier, Germany; e Research Center Smart Digital Health, University of the Bundeswehr Munich, GER, Germany

**Keywords:** Trier social stress test, Virtual reality, Cortisol, Alpha-amylase, Blood glucose, Heart rate, Stress response

## Abstract

**Background:**

The Trier Social Stress Test (TSST) is a widely used tool for inducing and measuring stress responses in a controlled environment. In this study, we aimed to explore the effectiveness of a virtual TSST (TSST-VR) in eliciting stress responses across multiple physiological and psychological markers.

**Methods:**

A sample of 24 participants underwent the TSST-VR, during which salivary cortisol, alpha-amylase (AA), blood glucose levels, heart rate (HR), root mean square of successive differences (RMSSD) as a measure of heart rate variability (HRV), and subjective stress ratings (NRS) were collected at multiple time points.

**Results:**

In a baseline-to-peak analysis, significant increases were observed in HR (M_Diff_ = 13.04, 95 %-CI [8.19–17.90], p < .001), RMSSD (M_Diff_ = 17.75, 95 %-CI [3.28–32.22], p < .001), AA (p < .001, r = 1.07), and NRS (p < .001, r = 1.31) measures following the TSST-VR. While no significant changes in cortisol levels were found in the baseline-to-peak analysis across all participants, a secondary cluster analysis identified distinct cortisol responders (baseline-to-peak rise >1.5 mmol/l). Within this group, high cortisol responders (HCR) showed significantly higher cortisol (Wald χ^2^(7) = 118.03, p < .001), HR (Wald χ^2^(8) = 17.91, p = .022), and AA levels (Wald χ^2^(7) = 17.13, p = .017) compared to low cortisol responders (LCR). Area-under-the-curve analysis further confirmed a more robust cortisol stress response in HCR.

**Conclusion:**

These findings suggest that the TSST-VR can effectively induce measurable stress responses and may provide insights into individual differences in physiological and metabolic stress reactions. The study highlights the potential of virtual stress paradigms in stress research and underscores the advantages of a virtual setting in terms of standardization and economic considerations.

## Highlights

• TSST-VR effectively induces autonomic stress responses similar to traditional TSST.
• Salivary alpha-amylase and heart rate confirm strong sympathetic nervous system activation.
• Blood glucose levels rise during peak stress, indicating metabolic activation.
• Cortisol responses vary, with high responders showing stronger physiological stress reactions.
• Individual differences in stress sensitivity influence HPA and SAM axis activation.

## Graphical abstract

**Figure:**
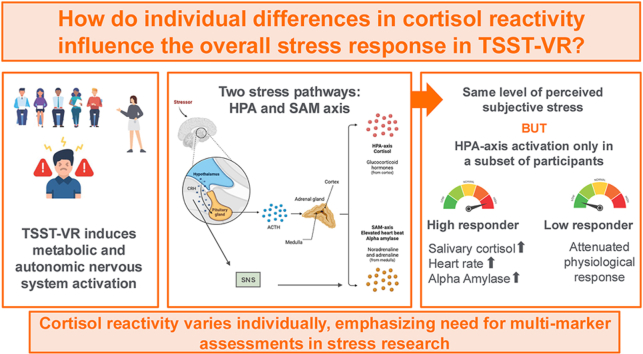

## 1 Introduction

Elevated heartbeat, sweating, arousal – telltale signs that the body is under stress. Acute stress triggers the body's reaction via two primary systems: The sympathetic-adrenal-medullary (SAM) axis and the hypothalamic-pituitary-adrenal (HPA) axis. Furthermore, acute psychosocial stress evokes a coordinated cascade across autonomic, endocrine, cardiovascular and metabolic systems: Within minutes, the sympathetic nervous system (SNS) engages, as evidenced by elevated heart rate (HR) and a decrease in heart rate variability (HRV), reflecting parasympathetic withdrawal. In the SAM axis, as a component of the broader SNS-mediated stress response, the release of adrenaline and noradrenaline in the adrenal medulla is stimulated, leading to an increase in salivary AA. The substantial increase of adrenaline in turn promotes glycogen breakdown in the liver and muscles, releasing glucose into the bloodstream. This results in elevated blood glucose levels to supply energy for the body's fight-or-flight response (Thews and Vaupel, 2005). To sustain the ongoing stress response, the slower and delayed activation of the HPA axis comes into play and releases cortisol, often referred to as the “stress hormone” (Smith and Vale, 2006). The secretion is initiated when the hypothalamus releases corticotropin-releasing hormone (CRH) into the bloodstream, stimulating the pituitary gland to secrete adrenocorticotropic hormone (ACTH). ACTH then prompts the adrenal cortex to produce glucocorticoids, primarily cortisol in humans. Plasma glucose levels are expected to rise during and after acute stress, because cortisol promotes gluconeogenesis and induces insulin resistance in muscle and fat tissue to preserve plasma glucose (Kuo et al., 2015). Higher blood glucose levels were found after stress exposure in patients with post-traumatic stress disorder (Nowotny et al., 2010) and Wiesli et al. showed increased blood glucose levels in patients with type 1 diabetes after acute psychological stress (Wiesli et al., 2005). Although these changes are adaptive for a brief bout of stress, if they persist beyond an acute episode they can have harmful metabolic consequences. Together, these tightly linked pathways ensure immediate and longer-term adaptation to social-evaluative threat. With the inclusion of blood glucose as a metabolic marker for stress, we tried to capture the full cascade from immediate SAM-axis turns to HPA-axis–mediated metabolic regulation, allowing us to test how stressors mobilize energy resources in parallel with autonomic and endocrine axes.

To study these physiological responses to stress, controlled laboratory stressors like the Trier Social Stress Test (TSST) are commonly used, a protocol that combines public speaking and mental arithmetic to induce social-evaluative stress (Kirschbaum et al., 1993). However, with recent advancements in virtual reality (VR), there is growing interest in exploring the potential of virtual stress testing environments to assess stress biomarkers more flexibly and ecologically.

The Virtual TSST (TSST-VR) offers a promising alternative to traditional in-person testing, providing participants with an immersive yet controlled environment to simulate real-world stressors (Standard et al., 2020). Unlike the standard TSST, which involves live human evaluators, the TSST-VR employs virtual avatars, maintaining the core elements of social-evaluative threat while allowing for greater standardization across trials. The use of VR can also address logistical challenges and participant accessibility, offering a more scalable solution for stress research. Recent studies show a growing trend in using VR to induce stress through various VR-adapted scenarios, such as simulated presentation tasks (Kothgassner et al., 2016a) or other stress-inducing settings (Dammen et al., 2022) and prior work has demonstrated that the TSST-VR successfully induces comparable cortisol and HR responses to its in-person counterpart (Jönsson et al., 2010; Zimmer et al., 2019a). Inter-individual variability in the bio-psycho-physiological reaction to the same stressor are known. Nater et al. (2007) were able to show differences between high and low cortisol responders, where high responders performed better on a declarative memory task after exposure to the TSST (Nater et al., 2007).

However, research on the TSST-VR remains relatively sparse, particularly with regard to its effects on multiple stress-related biomarkers and how these responses interact across systems.

Biomarkers such as cortisol, AA, HR, HRV and glucose levels are critical indicators of stress-induced changes in endocrine, cardiovascular and metabolic functions. Additionally, subjective stress ratings provide important insights into the individual's perceived stress experience, which may or may not align with physiological markers. While prior studies have explored these markers independently (Maron et al., 2016; Kothgassner et al., 2016b), few have examined how they concurrently respond to stress in virtual settings (Zimmer et al., 2019b; Gradl et al., 2019), nor how they may differ across high cortisol responders (HCR) and low cortisol responders (LCR) in the context of the TSST-VR.

In this study, we therefore aim to expand the understanding of the physiological and psychological responses to the TSST-VR by examining endocrine (cortisol, AA), metabolic (glucose), cardiovascular (HR), and psychological (self-reported stress) markers simultaneously. We hypothesize that the TSST-VR would elicit similar stress responses to those observed in the traditional TSST protocol established by Kirschbaum et al. (1993) (Kirschbaum et al., 1993) and repeated and extended by many others (For a meta-analysis see Helminen et al. (2021) (Helminen et al., 2021)). Secondly, we hypothesize that there might be participants who are more susceptible to a stressor than others (HCR) and that their cortisol response and reactions across other systems would be emphasized compared to their non-responding counterparts (LCR). The level of immersion, susceptibility to motion sickness, perception of social stress, or coping strategies employed during the virtual experience could be factors relevant to the perceived stress experience in the TSST-VR. This second hypothesis is based on the concept of Boyce et al. (1995) (Boyce et al., 1995) concept that stress reactivity is a unified, interconnected response involving cardiovascular, endocrine, and immune systems. It suggests that individuals that are reactive in one system are likely to exhibit similar reactivity across other systems as well.

By integrating these measures, this research seeks to clarify the relationships between various systems under virtual stress conditions and to explore the extent to which TSST-VR elicits comparable stress responses to the traditional TSST.

## 2 Materials and methods

### 2.1 Sample population

Participant recruitment was conducted through e-mail distribution and on-campus advertisement. The total sample population was limited to employees and students of the University of the Bundeswehr Munich (UniBw M) who were officers and officer candidates of the Bundeswehr. To determine the required sample size for an ANOVA with within-subject factors and eight repeated measurements, we conducted an a priori calculation in G*Power (Faul et al., 2007). Using an effect size of f = .65, based on a meta-analysis of salivary cortisol responses to VR-TSSTs (Helminen et al., 2019), the analysis indicated a minimum total sample size of N = 17 (α = .05, 1-β = .95). The recruited sample consisted of 28 participants, thereof 15 male and 13 female participants. Exclusion criteria included blood clotting disorders, use of medications affecting blood clotting, diagnosed hemophilia, type 1 or type 2 diabetes, acute or chronic infections, severe mental illnesses or post-traumatic stress disorder (PTSD), epilepsy, and chronic muscle twitching. Participants were informed about potential risks and signed a consent form. Due to issues encountered during data collection (faulty adhesion of the sensors to the subject and therefore, missing data acquisition), the final sample size was reduced to 24 participants, comprising 13 male and 11 female participants. We handled missing data via listwise deletion. The participants’ age ranged between 20 and 39 years, with an average age of 22.76 years (SD ± 3.78 years). See Table 1 for baseline participant characteristics.

**Table 1:** Baseline participant characteristics (total) and by cortisol responder status (HCR = High cortisol responder, LCR = Low cortisol responder) (mean ± SD or N) with p-values for group differences. Abbreviations: HR = Heart rate, AA = Alpha amylase, RMSSD = Root Mean Square of Successive Means.

Variable	HCR	LCR	Total	p-value
N (male:female)	11 (4:7)	10 (9:4)	21 (11:14)	.115
Age (years)	22.91 (±2.10)	22.70 (±5.39)	22.80 (±3.87)	.194
Glucose (mg/dl)	118.10 (±18.35)	118.31 (±18.48)	118.21 (±18.02)	.977
HR (bpm)	82.26 (±10.32)	76.19 (±15.99)	78.98 (±13.76)	.292
NRS	1.99 (±1.79)	1.23 (±1.59)	1.13 (±1.65)	.595
Cortisol (nmol/l)	3.31 (±2.57)	3.36 (±2.30)	3.33 (±2.37)	.505
AA (U/L)	155.03 (±87.71)	126.42 (±92.21)	139.53 (±89.40)	.447
RMSSD (ms)	40.55 (±26.67)	48.69 (±24.78)	44.96 (±25.43)	.235

### 2.2 Study design

The study procedures and measurements were approved by the ethics committee of the UniBw M. Three days before the TSST-VR testing, a continuous glucose monitoring system was attached on the participants’ upper arm to measure interstitial blood glucose concentrations.

**Fig. 1:**
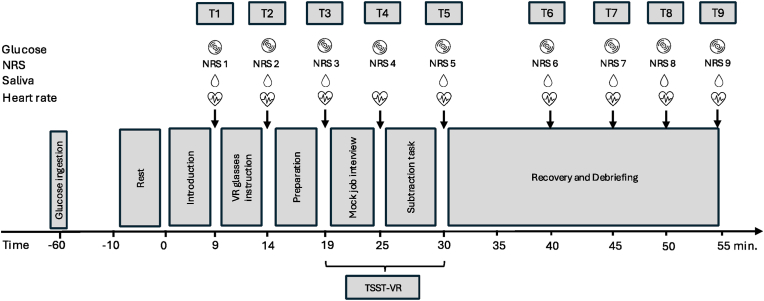
Study protocol and sampling times. Abbreviations: NRS: Numerical rating scale (subjective stress rating from 0 = no stress to 10 = extreme stress), TSST-VR: Virtual Trier Social Stress Test.

On testing day, the TSST-VR took place at the UniBw M in Neubiberg between 2pm and 7 p.m. Participants were assigned a 10-min rest period after arriving at the laboratory to restore their resting HR. Participants were instructed to refrain from nicotine, alcohol, sugar, and food for 1 h before the test, except for consuming 5.75 g of glucose (89 % dextrose) to ensure a consistent blood glucose level throughout the sample. During the rest period, compliance with these pre-test requirements was checked and the chest strap for HR monitoring was attached. Participants gave written informed consent and were briefed on the numerical rating scales (NRS) and salivary sample collection. During the experiment, eight saliva samples were collected. Additionally, at nine different time points, participants completed stress ratings on a numerical scale (0 = no stress, 10 = extreme stress) (NRS) to evaluate their subjective stress levels throughout the experimental procedures and HR was collected (see Fig. 1 for detailed sampling time frames).

We used the Open TSST-VR, an open source version of the virtual TSST protocol by Linnig et al. (2025) (Linnig et al., 2025). Participants were prepared for the VR scenario by fitting the HMD (Head Mounted Display) (Occulus Quest 2, Meta Platforms Inc., California, USA) and task instructions were given via headphones or written on screen, including preparation for a mock job interview to be presented to a panel of three judges. After the instructions, participants had 5 min to prepare. The job interview began at minute 20, during which the experimenter could interrupt or continue the session with questions from the panel. The interview ended at minute 25, without any feedback or affiliative behavior of the judges. Immediately afterward, participants transitioned to the second part of the TSST-VR, which involved a subtraction task, requiring them to subtract 17 from 2023 continuously. Any incorrect answers prompted a restart from 2023. After 5 min, the TSST-VR concluded, and participants removed the VR headset. The experimenter informed the participant that the VR scenario was complete and that the debriefing phase started, which included a 25-min rest period with reading material (journals and newspaper). Additional salivary samples, NRS recordings and HR sampling occurred at minutes 40, 45, 50, and 55.

### 2.3 Blood glucose levels

Blood glucose concentration was measured using the Freestyle Libre 3 Sensor (Abbott Laboratories, Illinois, USA). Data were recorded at 5-min intervals with a continuous Bluetooth Low Energy connection between the sensor and the Samsung Galaxy A15 research phone (Samsung Electronics Co., Ltd., Seoul, South Korea). The data were transmitted to an Abbott-configured cloud and read using Abbott's online application, LibreView. The collected data were aggregated in Microsoft Office 365 Excel (Version 2401, Microsoft Corporation, Redmond, USA).

### 2.4 HR and HRV recording

HR values and RR-intervals were recorded using a Polar Pro chest strap with a Polar H10 sensor (Polar Electro, Finland), connected to an Apple iPad mini (Apple Inc., Cupertino, USA). RR-intervals were calculated as the root mean square of successive differences (RMSSD; in ms) using the Kubios HRV - Daily Readiness application (Version 1.3.8) (Kubios Oy, Kuopio, Finland). The stored HRV data were exported and analyzed using the Kubios HRV Scientific application (Version 4.1.0). Low RMSSD values indicate reduced parasympathetic activity, corresponding to heightened physiological arousal, while high RMSSD values indicate enhanced parasympathetic activity, reflecting a state of lower physiological arousal.

### 2.5 Saliva sampling

Saliva samples were collected via Salivettes (Sarstedt, Nümbrecht, Germany) at eight time points – see Fig. 1. We deliberately omitted the saliva sample immediately after the mock interview (T4) to avoid interrupting the flow of the testing procedure and unduly prolonging the participant's experience. Participants were directed to hold the Salivette in their mouth for a minimum of 1 min, moving it side to side without chewing on it. Saliva samples were stored at −20° after collection. The analysis was conducted in the in-house laboratory of the Unibw M. Before analysis, the salivettes were thawed at room temperature and centrifuged (Medifuge, Thermo Fisher Scientific, Waltham, MA, USA) at 2000 g and 20 °C for 10 min immediately prior to testing. Salivary AA was measured with an enzyme kinetic assay using reagents from DiaSys Diagnostic Systems GmbH (Holzheim, Germany). Salivary cortisol concentrations were determined using chemiluminescence immunoassay (CLIA, IBL, Hamburg, Germany). Intra- and inter-assay coefficients of variation were 3.39 and 3.76 for AA and 4.21 and 5.08 for cortisol, respectively.

### 2.6 Numerical rating scale

A Numerical Rating Scale (NRS) (Karvounides et al., 2016) was used instead of a Visual Analogue Scale (VAS) due to the lack of visual assessment while wearing the VR headset. The subjective stress experience was recorded at nine defined time points during preparation and VR phases using the NRS, where participants rated their stress from 0 (no stress) to 10 (extreme stress).

### 2.7 Statistical analysis

Analyses of variance (ANOVA) for repeated measures were performed for HR and RMSSD to reveal possible time effects between baseline and peak values. Baseline value was defined as the lowest mean value before the peak (for RMSSD: the highest mean value before the lowest). Peak value was defined as the highest mean value of each parameter (for most markers at T5, post-arithmetic; for glucose at T4, post-interview; for cortisol at T7, 25 min after the onset of the stressor). We based our baseline-to-peak approach on the procedures of other VR-based studies that were summarized and analyzed in the meta-analysis from Helminen et al. (2019). In cases where distribution of normality was violated (AA, Cortisol, Glucose, NRS), a non-parametric test (Friedman-Test) was applied to determine possible differences between these time points. Greenhouse-Geisser adjustment was employed to account for violations of sphericity where appropriate. Post-hoc analyses were Bonferroni-adjusted. Statistical significance was accepted at p < .05. Effect sizes for Kendall's W and for pairwise comparisons (r) were calculated according to Cohen (1992): small (>.1), moderate (>.3), and large (>.5).

The sample was clustered in high cortisol responders and low cortisol responders based on the cortisol response criterion of a 1.5 nmol/l increase from baseline to peak, as proposed by Miller et al. (2013), to test the hypothesis, that elevated stress reactivity (cortisol increase) is associated with elevated values in other endocrine, physiological and metabolic parameters.

Generalized Estimating Equations (GEE) were employed to analyze repeated measures data and account for the correlated nature of measurements taken across multiple time points. GEE was chosen due to its ability to handle non-normal distributions and account for within-subject correlations, making it suitable for analyzing longitudinal data with repeated measurements. GEE models were fitted to examine the effects of time (measurement points) and group (HCR/LCR) on the dependent variables (glucose, HR, RMSSD, AA, NRS). Time was treated as a repeated factor, and group was included as a between-subject factor.

An exchangeable correlation structure was applied, assuming that the correlations between repeated measurements within each subject are constant over time. To evaluate the significance of main effects and interactions, Wald χ^2^ tests were conducted. Bonferroni corrections were applied to adjust for multiple comparisons where necessary. Furthermore, Area under the Curve respective to ground (AUC_g_) and Area under the Curve respective to increase (AUC_i_) for each group was computed to represent total cortisol output and the change in cortisol levels from baseline, respectively. Group differences were analyzed by *t*-test (AUC_i_) and Man-Whitney-U-test (AUC_g_). A p-value of < .05 was considered statistically significant. Data analysis was done with SPSS 29® (IBM SPSS, Armonk, NY, USA) and graphs were performed using R (version 4.3.2) software.

## 3 Results

### 3.1 Baseline-to-peak analysis

**Fig. 2:**
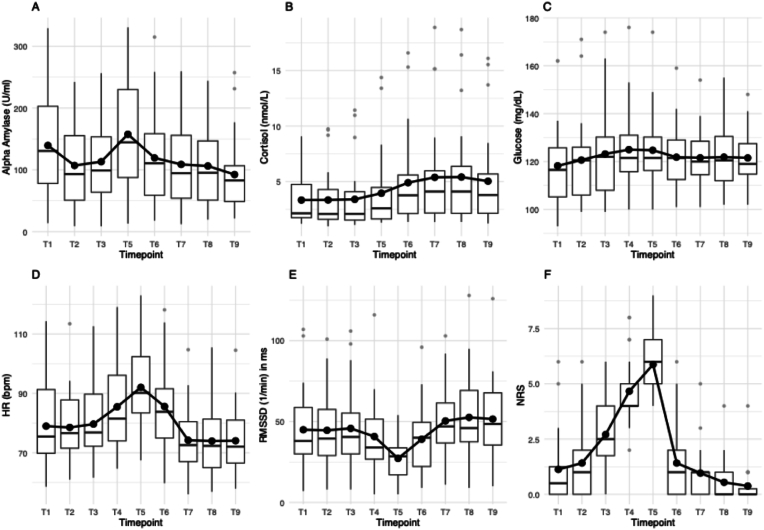
Distribution and mean values of alpha-amylase (A), cortisol (B), glucose (C), heart rate (D), RMSSD (E), and NRS (F) over the course of the experiment. Outliers are marked as grey dots. Abbreviations: RMSSD: root mean square of successive differences, NRS: numerical rating scale (subjective stress rating from 0 = no stress to 10 = extreme stress).

Fig. 2 displays distributions and mean values of all parameters. Mean HR was highest at T5. Repeated measures ANOVA determined that mean HR values showed a statistically significant difference between baseline to peak measurements (F(3.16, 72.64) = 41.94, p < .001). Post-hoc analysis revealed statistically significant (p < .001) higher HR measures in T5 than T1 (M_Diff_ = 13.04, 95 %-CI [8.19–17.90].

Mean RMSSD was lowest at T5. Repeated measures ANOVA determined that mean RMSSD values showed a statistically significant difference between baseline to peak measurements (F(3.38, 77.75) = 14,48, p < .001). Post-hoc analysis revealed statistically significant (p < .001) lower RMSSD measures in T5 compared to T1 (M_Diff_ = 17.75, 95 %-CI [3.28–32.22]).

Median Glucose was highest at T4. The Friedman-test revealed statistically significant differences across time points (χ^2^ (8) = 22.87, p = .004, W = .12). Differences were observed between T1 (Mdn = 3.46) and T4 (Mdn = 6.19) with p = .02, r = .70).

Median NRS was highest at T5. The Friedman-test revealed statistically significant differences across time points (χ^2^ (8) = 155.43, p < .001, W = .81). Differences were observed between T1 (Mdn = 3.75) and T5 (Mdn = 8.33) with p < .001, r = 1.31).

Median AA was highest at T5. The Friedman-test revealed statistically significant differences across time points (χ^2^ (7) = 48.14, p < .001, W = .29). Differences were observed between T2 (Mdn = 3.38) and T5 (Mdn = 7.08) with p < .001, r = 1.07).

Median Cortisol was highest at T8. However, no statistically significant differences were found in the cortisol concentrations across time points, χ^2^(7) = 12.03, p = .10, W = .07.

### 3.2 Group and time differences: high cortisol responders/low cortisol responders

**Fig. 3:**
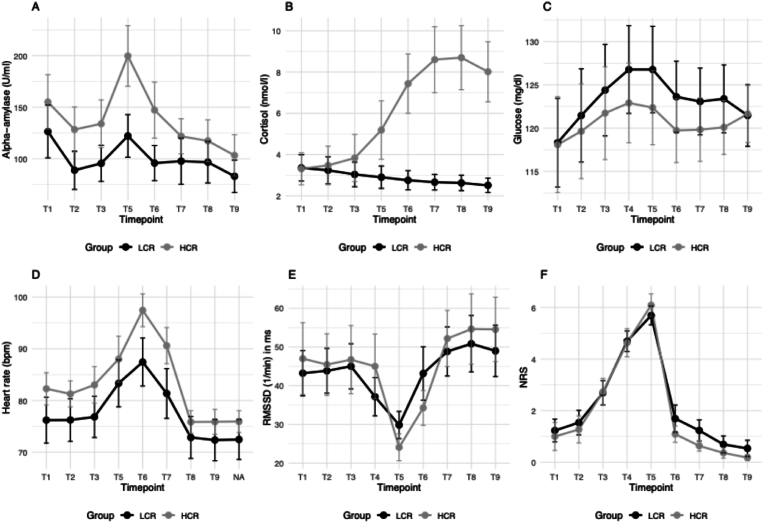
Dynamics of alpha-amylase (A), cortisol (B), glucose (C), heart rate (D) RMSSD (E) and NRS (F) over the course of the experiment and clustered in high and low cortisol responders. Abbreviations: RMSSD: root mean square of successive differences, NRS: numerical rating scale (subjective stress rating from 0 = no stress to 10 = extreme stress).

11 (46.0 %) out of 24 participants were categorized as HCR according to the cortisol response criterion of 1.5 nmol/l increase from baseline to peak (Fig. 3).

Sex and age distributions did not differ by responder status (sex: Fisher's exact p = .217; age: U = 50.0, p = .23). Likewise, there were no baseline group differences on any physiological or subjective measure between groups (glucose t(22) = .03, p = .98; HR t(22) = –1.08, p = .29; AA: t(22) = –.77, p = .45; cortisol U = 60.0, p = .53; NRS: stress U = 63.0, p = .65; RMSSD: U = 51.0, p = .25).

The GEE analysis revealed a significant interaction between group and time on AA secretion (Wald χ^2^(7) = 17.13, p = .017). Pairwise comparisons indicated that HCR showed significantly higher AA levels compared to LCR at time points T4 and T5 (p = .012 and p = .015, respectively). The main effect of time was also significant (Wald χ^2^(7) = 73.93, p < .001), indicating an overall change in AA levels over the measurement period.

A significant interaction between group and time was observed for HR (Wald χ^2^(8) = 17.91, p = .02), with HCR showing higher HR compared to LCR at T5 (p = .004). The main effect of time was also significant (Wald χ^2^(8) = 295.14, p < .001), suggesting a time-dependent increase in HR for both groups.

No significant group and time interaction effects were found for subjective stress ratings on the NRS (Wald χ^2^(8) = 5.28, p = .73), glucose (Wald χ^2^(8) = 7.33, p = .50), and RMSSD (Wald χ^2^(8) = 7.38, p = .49), suggesting that HCR and LCR did not differ in their subjective stress perception, glucose secretion, and parasympathetic activity over time.

### 3.3 Cortisol levels and AUC_g_ and AUC_i_

A significant main effect of group was observed for cortisol levels, indicating that HCR and LCR exhibited different cortisol profiles across the study, Wald χ^2^(1) = 6.19, p = .013. Additionally, there was a significant main effect of time, Wald χ^2^(7) = 75.76, p < .001, reflecting significant variations in cortisol levels across the measurement points. Furthermore, a significant interaction effect between group and time was found, Wald χ^2^(7) = 118.03, p < .001, suggesting that the pattern of cortisol release over time differed between HCR and LCR.

To further explore the cortisol response, both AUC_G_ and AUC_i_ were calculated for HCR and LCR. Independent t-tests showed that HCR had significantly higher AUC_i_ values (M = 119.57, SD = 84.04, overall AUC_i_ = 1315.3) compared to LCR (M = −21.30, SD = 29.05, overall AUC_i_ = −276.9), t(22) = −5.67, p < .001. This indicates that HCR exhibited a higher increase in cortisol levels across the study period.

**Fig. 4:**
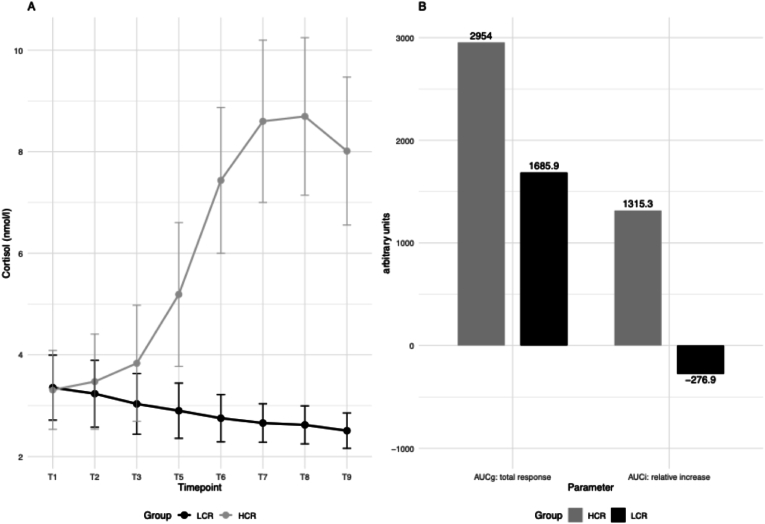
Concentration of free salivary cortisol in response to the TSST-VR sampled at eight time points over the course of the experiment and clustered in high cortisol responders (baseline to peak increase: >1.5 nmol/l) and low cortisol responders (A). Total cortisol response (AUC_g_) and relative increase (AUC_i_) during the stress experiment (B). Abbreviations: AUC_i_: Area Under Curve with respect to increase, AUC_g_: Area Under Curve with respect to ground.

For AUC_G_, results showed a significant difference between groups, with HCR secreting a greater overall amount of cortisol (Mdn = 16.55, overall AUC_G_ = 2954.0) compared to LCR (Mdn = 9.08, overall AUC_G_ = 1685.9), U = 116.00, z = 2.58, p = .009. These findings suggest that while both groups experienced fluctuations in cortisol over time, the overall amount of cortisol (AUCg) and the dynamic response to stress (AUCi) were significantly greater in HCR (Fig. 4).

## 4 Discussion

This study aimed to examine whether endocrine, cardiovascular, and psychological stress markers in the TSST-VR align with those in the traditional in-person TSST. We also sought to identify cortisol responder subgroups based on individual HPA axis reactivity.

We hypothesized that the TSST-VR would elicit comparable physiological and subjective stress responses to the original TSST, and that HCR would show elevated biopsychological reactivity across multiple parameters compared to LCR.

Our results revealed significant increases in HR, salivary AA, glucose, and subjective stress ratings, along with a decrease in HRV following the TSST-VR. However, no significant overall cortisol increase was observed. Subgroup analysis identified 11 out of 24 participants as HCR. This group exhibited significant cortisol increases, as well as elevated HR and AA levels, compared to the LCR group, who showed little to no cortisol response.

Our findings support the notion that the TSST-VR can successfully induce acute stress responses. Peak levels of HR and AA, along with the lowest values of RMSSD, were observed at T5 following the arithmetic task—reflecting clear activation of the sympathetic nervous system and being consistent with its well-established role in acute stress responses (‘fight-or-flight’ (Cannon, 1929)). Subjective stress ratings followed a similar trajectory, peaking concurrently with physiological markers, further confirming the test's stress-inducing capacity. Blood glucose levels peaked after the mock job interview and before the subtraction task, suggesting anticipatory metabolic activation. This response likely reflects SAM-axis-driven adrenaline release, which promotes glycogenolysis and rapidly increases blood glucose to meet the energy demands of acute stress. Our findings suggest that elevated glucose levels supported metabolic readiness during the most intense phase of the TSST-VR. While no significant baseline-to-peak increase in cortisol was observed in the overall sample, subgroup analysis revealed that cortisol concentrations peaked significantly 20 min after the task in HCRs, whereas the LCR group showed no notable cortisol changes.

These results are consistent with existing research on TSST and TSST-VR, as such increases are typically observed during the most stressful phases of the protocol (public speaking and arithmetic tasks) which have been well documented in both traditional TSST studies (Kirschbaum et al., 1994; Kudielka et al., 2004) and VR-based scenarios (Linnig et al., 2025). Salivary AA is well established in the literature as a marker of SAM axis activity, peaking rapidly after stress exposure (Nater and Rohleder, 2009). Cardiovascular changes in response to acute stressors, including elevated HR, have also been repeatedly demonstrated in traditional TSST studies, with responses beginning at stress onset and continuing for up to 30 min post-task (Allen et al., 2014). Reduced RMSSD values, as seen at T5, indicate diminished parasympathetic activity and heightened physiological arousal (Kim et al., 2018). Supporting our findings, Shiban et al. (2016) observed increased self-reported stress and HR across different TSST modalities, including VR versions (Shiban et al., 2016). Furthermore, our results align with prior VR-based research that has consistently shown robust autonomic and subjective responses, but lower and more attenuated cortisol reactivity compared to in-person TSST (Dammen et al., 2022; Helminen et al., 2019). The study by Kelly et al. (2007) suggests that virtual environments, particularly those involving virtual audiences, are associated with weaker cortisol responses (Kelly et al., 2007). These effects may be influenced by factors such as participant age, physical training status (Rimmele et al., 2007, 2009), and the level of immersion in the VR environment (Helminen et al., 2019).

In line with Kirschbaum et al. (1995), our results provide evidence for two distinct cortisol response profiles: high cortisol responders and low cortisol responders (Kirschbaum et al., 1995). Subgroup analysis identified 46 % of all participants as high cortisol responders, exhibiting significant cortisol increases, along with elevated HR and AA levels, compared to the LCR group. This pattern was confirmed by area-under-the-curve analyses (AUCg and AUCi), indicating a more dynamic and sustained HPA axis response in the HCR group. The absence of a cortisol increase in LCR does not necessarily indicate a lower stress load. Studies have shown that blunted cortisol responses may be associated with increased risk for dysfunctional HPA regulation (Silverman and Sternberg, 2012). On the other hand, persistent high cortisol responses, as seen in hypercortisolism, have been linked to Major Depressive Disorder (Dean and Keshavan, 2017). Moreover, existing research suggests that cortisol responsiveness is modulated by various individual factors including sex, age, mood and personality traits (Kudielka et al., 2009).

Interestingly, both subgroups reported similar levels of subjective stress, despite pronounced differences in physiological responses. This dissociation has been noted in the literature. Campbell and Ehlert (2012) found that only about 25 % of studies report significant correlations between perceived stress and physiological responses, suggesting that emotion regulation, cognitive appraisal, coping strategies, and personality traits can influence this relationship (Campbell and Ehlert, 2012). Renner et al. identified conscientiousness as a moderator of this alignment, with more conscientious individuals showing a stronger match between subjective stress perception and cortisol reactivity (Talić et al., 2023).

Coping strategies may also play a role in modulating physiological responses. Studies suggest that individuals engaging in active, problem-focused coping exhibit lower cortisol levels, indicating that subjective stress alone does not predict biological reactivity (Bohnen et al., 1991; O'Donnell et al., 2008). This may explain why participants in the LCR group experienced perceived stress without corresponding physiological activation.

Nevertheless, our results do not allow for a clear classification into either HPA or SAM axis activation. As mentioned before, the activation of the SAM axis is associated with acute, short-term stress responses, whereas the HPA axis contributes to longer-term effects. Both axes are part of a closely linked stress system and are generally thought to work in a coordinated or sequential pattern to adapt to stressors (Wadsworth et al., 2019; Godoy et al., 2018). This means that strong activation in one system is usually accompanied by strong activation in the other—as seen in our HCR group. However, the two systems can also react differently depending on the nature of the stressor, its perceived controllability and the social-evaluative threat. While the HPA axis may be more sensitive to emotions like fear or frustration, the autonomic nervous system tends to respond more broadly to all kinds of stressors (Frankenhaeuser et al., 1986; Lovallo and Thomas, 2000).

Our study confirms that the TSST-VR can induce acute psychosocial stress, as indicated by SAM-axis activation in all participants and HPA-axis activation in a subset. However, several limitations must be considered. Cortisol was the only stress reactivity variable that was considered as it was the most used physiological stress reactivity variable in the TSST-VR literature and because of its central coordinating role in the stress response. While cortisol reactivity can drive endocrine, metabolic and subjective changes, bidirectional and system–system feedback also exists. To move beyond univariate or simple “cortisol-predicts-other” models, future studies might consider multivariate techniques (e.g., structural equation modeling (Milrad et al., 2018) or network analyses) to capture bidirectional and network‐level interactions among cortisol, AA, HR/HRV, and glucose.

The sample size was small, and we did not include a separate VR-control condition in this study, which limits our ability to rule out non‐specific VR effects (e.g. novelty or simulator discomfort) and to conclude that the stress response is solely based on the socio-evaluative threat and uncontrollability of the TSST scenario. However, in a study done by Zimmer et al. (2019a), physiological stress markers rose only in the evaluative VR‐TSST condition and not in the VR-control condition (talk about a self-chosen topic in an empty room) - indicating that mere immersion in an unfamiliar virtual setting is insufficient to trigger an HPA‐axis response.

Moreover, we did not assess participants’ prior VR experience, which may have influenced stress responses. Future research should address these limitations by incorporating larger and more diverse samples, placebo conditions, and systematically collecting data on immersion and VR familiarity.

In conclusion, our study delivers a first step towards multidimensional VR-TSST stress analysis - capturing endocrine, cardiovascular, metabolic and subjective stress responses. We also, for the first time in VR, identify high- and low-cortisol responders and reveal their distinct HPA, SAM, and metabolic reaction patterns. This work opens new insights into individual stress dynamics and the immediate coupling of energy mobilization to psychosocial threat in virtual environments.

## CRediT authorship contribution statement

**Eva Fellinger:** Writing – review & editing, Writing – original draft, Visualization, Formal analysis, Data curation. **Tom Brandt:** Writing – review & editing, Methodology, Investigation, Conceptualization. **Andrea Schittenhelm:** Investigation. **Eric Quarg:** Investigation, Formal analysis, Data curation. **Matthias Pröll:** Investigation, Formal analysis, Data curation. **Gregor Domes:** Writing – review & editing, Software. **Annette Schmidt:** Writing – review & editing, Methodology, Conceptualization.

## Ethics approval and consent to participate

The study was conducted according to the guidelines of the Declaration of Helsinki and approved by the Ethics Committee of the University of the Bundeswehr Munich, Germany (EK UniBw M 25-16). From all participants consent was obtained before participation in the study.

## Transparency statement

Eva Fellinger affirms that this manuscript is an honest, accurate, and transparent account of the study being reported; that no important aspects of the study have been omitted; and that any discrepancies from the study as planned (and, if relevant, registered) have been explained.

## Funding

We acknowledge financial support by Universität der Bundeswehr München.

## Declaration of competing interest

The authors declare the following financial interests/personal relationships which may be considered as potential competing interests: Eva Fellinger reports financial support was provided by Universität der Bundeswehr München. If there are other authors, they declare that they have no known competing financial interests or personal relationships that could have appeared to influence the work reported in this paper.

## Data Availability

Data will be made available on request.
